# A review of HSV pathogenesis, vaccine development, and advanced applications

**DOI:** 10.1186/s43556-024-00199-7

**Published:** 2024-08-29

**Authors:** Lan Bai, Jiuzhi Xu, Linghui Zeng, Long Zhang, Fangfang Zhou

**Affiliations:** 1 International Biomed-X Research Center, Second Affiliated Hospital of Zhejiang University School of Medicine, Zhejiang University, Hangzhou, 310058 China; 2 Center for Oncology Medicine, the Fourth Affiliated Hospital of School of Medicine, and International School of Medicine, International Institutes of Medicine, Zhejiang University, Yiwu, 322000 China; 3 Zhejiang Key Laboratory of Precision Diagnosis and Treatment for Lung Cancer, Yiwu, 322000 China; 4 School of Medicine, Zhejiang University City College, Hangzhou, 310015 China; 5 MOE Laboratory of Biosystems Homeostasis & Protection and Innovation Center for Cell Signaling Network, Life Sciences Institute, Zhejiang University, Hangzhou, China; 6 Cancer Center, Zhejiang University, Hangzhou, China; 7 Institutes of Biology and Medical Science, Soochow University, Suzhou, 215123 China

**Keywords:** Herpes simplex virus, Pathogenesis, Immune evasion, Vaccine, Biological application

## Abstract

Herpes simplex virus (HSV), an epidemic human pathogen threatening global public health, gains notoriety for its complex pathogenesis that encompasses lytic infection of mucosal cells, latent infection within neurons, and periodic reactivation. This intricate interplay, coupled with HSV's sophisticated immune evasion strategies, gives rise to various diseases, including genital lesions, neonatal encephalitis, and cancer. Despite more than 70 years of relentless research, an effective preventive or therapeutic vaccine against HSV has yet to emerge, primarily due to the limited understanding of virus-host interactions, which in turn impedes the identification of effective vaccine targets. However, HSV's unique pathological features, including its substantial genetic load capacity, high replicability, transmissibility, and neurotropism, render it a promising candidate for various applications, spanning oncolytic virotherapy, gene and immune therapies, and even as an imaging tracer in neuroscience. In this review, we comprehensively update recent breakthroughs in HSV pathogenesis and immune evasion, critically summarize the progress made in vaccine candidate development, and discuss the multifaceted applications of HSV as a biological tool. Importantly, we highlight both success and challenges, emphasizing the critical need for intensified research into HSV, with the aim of providing deeper insights that can not only advance HSV treatment strategies but also broaden its application horizons.

## Introduction

The herpes simplex virus (HSV), a highly prevalent human pathogen with a global seroprevalence of 66%, comprises type 1 (HSV-1) and type 2 (HSV-2), primarily linked to orofacial and genital lesions respectively [8, 9]. HSV infections are ubiquitous and covert, silently targeting mucosa and skin of all ages, and remaining latent in neurons for life. Approximately 80% of patients remain asymptomatic during the lytic infection, which leads to the narrow treatment window often being missed and the virus being unintentionally transmitted to partners or neonates, further exacerbating its spread [11–13]. Annually, approximately 14,000 neonatal infections worldwide originate from infected genital secretions during birth, leading to severe conditions such as neonatal encephalitis, pneumonia, and hepatitis, resulting in significant morbidity and mortality [14]. Unfortunately, HSV significantly elevates the risk of human immunodeficiency virus (HIV) infection by 3-4 times by eliciting an immune response within the genital tract that increases CCR5 receptor expression on CD4+ T cells, a key factor associated with HIV transmission [15, 16]. HSV is also implicated in various cancers like cervical squamous cell carcinoma and adenocarcinoma, oral cancer and prostate cancer, as well as age-related disorders such as type 2 diabetes and neurodegenerative diseases [21–27].

To date, there are no available cures or vaccines against HSV infections, making combating the disease and limiting its spread a challenge. However, medications such as acyclovir, valacyclovir, and ganciclovir have been widely used to control the symptoms, such as reducing viral shedding and shortening symptom duration [29]. Nevertheless, these only provide temporary relief as outbreaks can recur due to viral reactivation [30, 31]. Over the past few decades, numerous studies have been conducted to develop a vaccine to prevent or treat HSV infections, which would also potentially decrease HIV and human papilloma virus (HPV) infections, yet an ideal vaccine candidate remains elusive [16, 32]. This dilemma primarily arises from the intricate nature of HSV's pathogenesis and immune evasion mechanisms, which employ diverse pathways to evade host antiviral immune responses, presenting formidable challenges in identifying effective vaccine targets capable of eliciting and maintaining a robust immune response [15, 33, 34].

Nevertheless, looking at both sides of the coin, HSV's unique biological characteristics not only hinder profound research and vaccine development but also show immense promise as a versatile tool in clinical and scientific research. To be specific, HSV boasts a robust genetic load capacity, which endows it with potential applications in oncolytic virotherapy, gene therapy, and biological imaging [10, 35–39]. Its neurotropism is crucial, enabling it to play a central role in the investigation of neuronal disorders [40, 41]. HSV has also been utilized in vaccine carrier development and disease modeling. However, despite its vast potential for applications, many inherent risks and challenges persist, such as targeting and gene expression regulation abilities [36, 43]. Thus, continued research into HSV is essential for gaining a comprehensive understanding of the virus, addressing existing limitations, and advancing the development of vaccines and applications.

In this review, we focus on HSV pathogenesis, encompassing biological characteristics, lytic and latent infections, reactivation, and virus-host immune interactions, with the aim of providing a thorough overview and in-depth insights that will contribute to the advancement of vaccine development for disease control and HSV-based tools. Additionally, we examine the recent progress in vaccine candidates and potential applications of HSV-based biological tools, highlighting their successes and challenges, with the aim of contributing to achieving control over HSV infection and harnessing its full potential as a versatile tool.

## Pathogenesis of HSV

### Structural and biological characteristics

HSV is a double-stranded, DNA-enveloped virus. The DNA core is enclosed within an icosahedral protein capsid comprising 162 capsomeres that form a viral particle. The particle is further surrounded by a protein-rich unstructured matrix called a tegument, which contains several proteins associated with viral replication and immune evasion (VP16, UL36, and VP22). Enveloping the tegument is a lipid bilayer membrane decorated with 13 branched glycoproteins, including glycoproteins B, C, D, and E, that play crucial roles in viral invasion and immune evasion [44–48].

The HSV-1 genome consists of approximately 150 kilobase pairs (kbp); however, 284 open reading frames (ORFs) have recently been defined, which contradicts the previous results of 80 ORFs [49]. This significant difference underscores the immense complexity of the HSV genome and protein expression, posing significant challenges in understanding its pathogenesis and developing effective vaccines. Nevertheless, this also hints at the immense genetic load capacity of the HSV genome. The viral mRNA is synthesized by sequential transcriptional cascades under the regulation of viral factors, with the participation of the host cell RNA-polymerase II. HSV genes are categorized into three groups based on their expression order: immediate early genes (IE or α genes), early genes (E or β genes), and late genes (L or λ genes) [50, 51]. IE genes are the first genes whose expression is regulated by tegument protein VP16. The products of IE genes are closely associated with lytic viral and latent infections. For instance, ICP0 and ICP4 play important roles in viral replication and expression of the E and L genes. E genes encompass β1 and β2 proteins, with β2 proteins mainly being responsible for viral nucleic acid metabolism, including thymidine kinase and DA polymerase. L proteins comprise viral structural proteins, such as glycoproteins, capsid proteins, and endometrial proteins, which are integral to viral attachment, entry, and antigenicity [52].

### Lytic infection

#### Host-cell entry

**Fig. 1:**
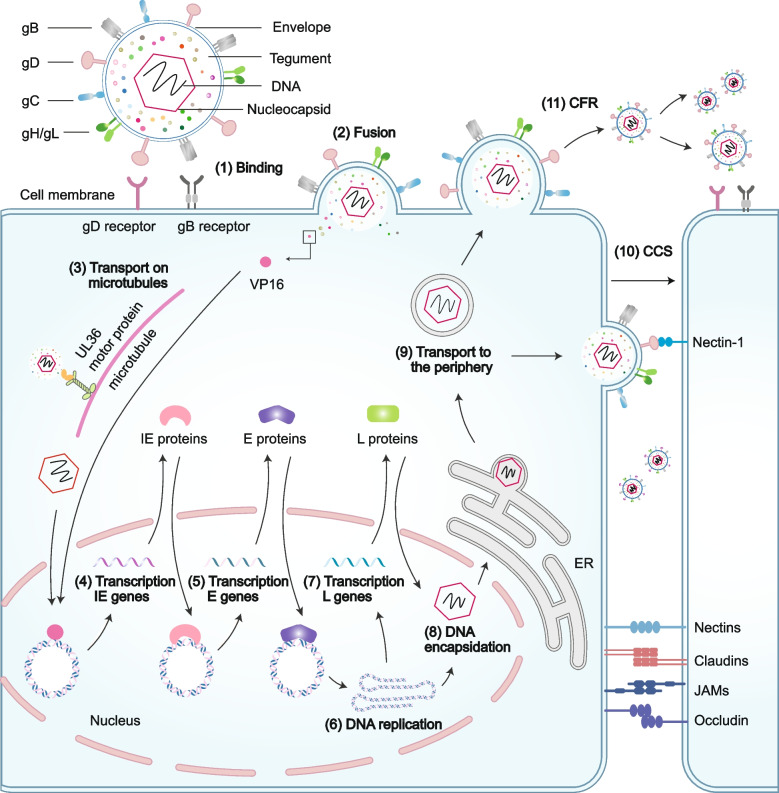
Host cell entry and transmission of HSV. (1) HSV gD interacts with a specific cellular receptor, initiating a cascade that activates gH and gB (not shown). (2) gB leads to fusion at the plasma membrane. (3) The capsid, accompanied by tegument proteins, is released into the cytoplasm, where it traverses to the cell nucleus along microtubules, which is facilitated by the interaction between UL36 and motor proteins. Subsequently, the linear DNA is released into the nucleus and converted into a circular genome. (4) Meanwhile, tegument protein VP16 detaches from the capsid and independently enters the nucleus, where it starts the transcription of IE genes by recruiting host cell factors. (5) The IE genes are translated and participate in the transcription of E genes. (6-7) The E genes are translated and take part in the replication of the viral genome and start transcription of L genes. (8) Once a sufficient number of viral genome copies are attained, the products of the L genes aid in the process of DNA encapsidation. (9) The mature virus leaves the nucleus via an envelopment-deenvelopment process, acquiring tegument and envelope (not shown) prior to cellular egress. (10-11) Two major modes of HSV transmission, a virion within a vesicle traffics either to the cell surface and extracellular space, for CFR to infect neighboring or distant cells by recognizing specific receptors, or to the cell-cell junctions, for CCS to target adjacent cells via receptors or other factors, for release by exocytosis. CCS, cell-cell spread; CFR, cell-free release

Primary infections typically target the mucosal epithelium, where many progeny are produced and released through cell lysis. Host cell entry mainly involves two pathways: post-attachment fusion (Fig. 1) and endocytosis and phagocytosis-like uptake, both of which involve multiple viral glycoproteins. During post-attachment fusion, the virus rides along the filopodia surface of the target host cell, facilitated by the binding of glycoproteins B and/or C (gB and/or gC) to heparan sulfate proteoglycans (HSPGs). This allows the virus to reach the cell surface, where viral glycoprotein D (gD) attaches to one of its specific receptors, thereby marking the attachment step [9, 20]. The reported gD cellular receptors fall into three classes: herpes virus entry mediator (HVEM), nectin-1, nectin-2, and 3-O-sulfated heparan sulfate (Table 1). They are expressed in different cell types and act on various viral species [20, 28, 53]. Filopodia, key structures on various cell surfaces, express HSPG, increasing the likelihood of HSV attachment and enhancing infectivity [54–56]. Once attachment occurs, a signal is relayed to the glycoprotein H-L complex (gH-gL), activating gH and triggering a conformational change in the fusion gB from its pre-fusion to post-fusion state. Activated gB is inserted into the cell membrane, and facilitates the fusion of cells and viral membranes, followed by the creation of a pore in the cell membrane, which allows the viral capsid to enter the cytoplasm by refolding [9, 57–59]. Other studies have shown that gB initiates membrane fusion by binding to its receptors [54]. The gB receptors have three types: paired immunoglobulin-like type 2 receptor-α, an inhibitory receptor located on macrophages, dendritic cells (DCs), and monocytes; a myelin-associated glycoprotein present on glial cells; and non-muscle myosin heavy chain II found on human tissues (Table 1). Alternatively, HSV can enter host cells via endocytosis and phagocytosis-like uptake. HSV specifically binds to the gD receptor localized in the endosome, activates Rho GTPase, and rearranges the cytoskeleton, ultimately leading to fusion with the endosomal membrane [54, 60].

**Table 1:** HSV gD and gB receptors

Glycoprotein	Receptor	Function	Distribution
gD	Herpesvirus entry mediator (HVEM)	Almost all the clinical strains of HSV-1 and HSV-2 tested binds efficiently with HVEM [2].	Immune cells (e.g. T cells, B cells, DCs, NK, macrophages, polymorphonuclear cells), neurons, epithelial cells, fibroblasts [17].
	Nectin-1	Almost all HSV-1 and HSV-2 strains can bind to nectin-1 for entry [18, 19].	Epithelial cells, endothelial cells, neuronal cells, keratinocytes [20].
	Nectin-2	The nectin-2 is a receptor only for some mutant forms of HSV-1 and HSV-2.	Epithelial cells, endothelial cells, neuronal cells.
	3-O-sulphate-modified heparan sulphate (3-O-S HS)	It mediates entry of only HSV-1, but not HSV-2 [20].	Some selected human cell lines (e.g. endothelial and mast cells) and human tissues [18].
gB	Paired immunoglobulin-like type 2 receptor-α (PILRα)	It mediates entry of HSV-1, but not HSV-2 [28].	Immune cells, especially myeloid cells: monocytes, macrophages, dendritic cells [20].
	Myelin-associated glycoprotein (MAG)	It mediates entry of HSV-1.	Glial cells, not expressed in epithelial cells.
	Non-muscle myosin heavy chain II (NMHC-IIA, NMHC-IIB)	It mediates entry of HSV-1 [42].	NMHC-IIA: epithelial cells, endothelial cells, and neurons, NM-IIB: neuronal tissue [42].

As previously stated, filopodia formation and HSPG play crucial roles in HSV reaching the cell surface, gD mediates viral attachment or direct membrane fusion, and the heterodimer gH-gL and viral fusion gB are a set of core entry glycoproteins that are conserved in all herpes viruses. In summary, these proteins are required for cell entry. Blocking the virus at the entry step is a beneficial and broad strategy for developing vaccines and antiviral drugs, and several types of HSV substances targeting these targets have been developed, such as anti-HSPG peptides, anti-HSV.

antibodies, vaccines targeting glycoproteins, and inhibitors [54]. However, it is necessary to uncover the underlying cellular and molecular mechanisms of HSV infection and filopodia generation to better understand viral pathogenesis and promote the development of novel therapeutic strategies and more effective viral tools.

#### Genome expression

After the viral capsid and tegument enter the target cytoplasm, inner tegument proteins collaborate with host actin and myosin to facilitate the retrograde transport of the viral capsid along microtubules towards the nuclear pore, where the viral genome is released into the nucleus through rearrangement of the capsid proteins. Upon nuclear entry, viral DNA is promptly coated with histones and cellular repressors, serving as anchors for the assembly of nuclear domain 10 (ND10) bodies, which silence viral DNA. To fully express its function, HSV must overcome host suppression. Remarkably, the outer tegument proteins VP16, VP22, and pUL36 independently migrate to the nucleus before the genome, initiating viral DNA expression [47, 51, 61–66]. As mentioned above, viral genes are expressed in a cascade because of sequential derepression, evolved to maximize viral yield while minimizing host interference with viral DNA and protein synthesis, viral assembly, and elimination from the infected cells [67].

**Fig. 2:**
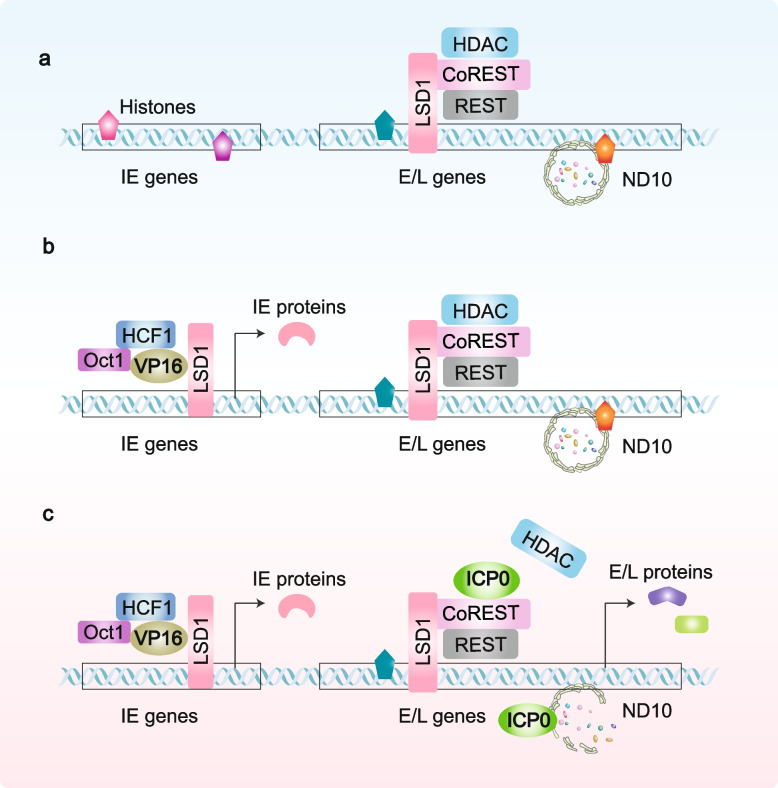
Genome expression. **a** The linear viral DNA is released into the nucleus and is promptly silenced by histones, HCLR repressor, and ND10 bodies. **b** The tegument protein VP16 recruits host factors HCF1, Oct-1, and LSD1 to their promoters to initiate the transcription of IE genes, including ICP0, ICP4, ICP22, and ICP27. **c** The IE protein ICP0 disrupts HCLR repressor by binding to CoREST and displacing HDAC, and degrades ND10 bodies via RING-finger E3 ubiquitin ligase activity, thereby leading to the releases and transcription of E and L genes. HCF1, host cell factor 1; Oct-1, octamer binding protein 1; LSD1, lysine specific demethylase 1; HDAC, histone deacetylase; CoREST, Co-RE1 silencing transcription factor; ND10, nuclear domain 10

IE genes are derepressed when VP16 recruits host cell factor 1 (HCF1), octamer binding protein 1 (Oct-1), and lysine -specific demethylase 1 (LSD1) to their promoters. This recruitment initiates the transcription of IE genes, including *ICP0*, *ICP4*, *ICP22*, and *ICP27*, via the cellular transcriptome (Fig. 2a) [68–70]. Moreover, the E and L genes are suppressed by ND10 bodies and the HCLR repressor complex, which is composed of histone deacetylases (HDAC1 or HDAC2), Co-RE1 silencing transcription factor (CoREST), LSD1, and REST. ICP0, however, disrupts this repression mechanism by binding to CoREST and displacing HDAC, leading to the release and transcription of E and L genes. Notably, ICP0, which possesses RING-finger E3 ubiquitin (Ub) ligase activity, can also degrade ND10 bodies (Fig. 2b,c) [71]. To express genes, viral DNA must be derepressed by modification of repressive histones, cellular repressors, and ND10 coating the viral DNA; VP16 and ICP0 are two key viral players.

ICP0 in the cytoplasm not only directly modulates genome expression but also facilitates viral replication by blocking host responses, such as the inactivation of IRF3 [72]. Furthermore, studies have revealed that pUL16 and pUL21 interact with nuclear pore complexes to interfere with capsid docking, which may prevent the production of progeny virions [73]. Currently, no antiviral drugs targeting ICP0, VP16, VP22, or UL21 exist. Nevertheless, studying these targets from diverse perspectives is crucial for enhancing our understanding of their mechanisms and facilitating the development of vaccines. Additionally, it aids in the precise regulation of exogenous gene expression during gene therapy.

#### Transmission

Progeny virions egress from the host cell and continue to infect new target cells via two pathways: cell-cell spread (CCS) and cell-free release (CFR) [74–77]. In CCS, viral particles are directly delivered to the cellular junctions to target adjacent cells (Fig. 1). The CCS tends to infect highly polarized cells, such as epithelial cells, as it guards virions against neutralizing antibodies and other soluble immunological factors. The highly directed nature of CCS likely enhances infection efficiency [74, 78]. In contrast, CFR involves the release of viral particles into the extracellular space, allowing them to travel and infect neighboring or distant cells by recognizing specific receptors (Fig. 1). CFR channels are critical for viral transmission between distant cells and hosts. Nevertheless, similar to other numerous enveloped viruses, HSV primarily relies on the efficiency and protective nature of CCS to disseminate the infection [79].

Although the precise mechanism of HSV transmission among cells remains unknown, studies have shown that several viral and host proteins are required [80–82]. Once the viral particle attains infectivity, that is, the nucleocapsid is enveloped in a vesicle, the vesicle will signal the cell periphery, either to the cell-cell junction for CCS or to the cell surface for CFR, where the mature virion is released through exocytosis [79]. In CCS, the progeny are released into the cell-cell junction, facilitating efficient interaction with host entry receptors. Nectin-1, a major entry receptor for gD, accumulates at these junctions, thereby promoting CCS. Notably, nectin-1 also functions as a host-adhesion transmembrane protein that mediates cellular adhesion (Fig. 1). This suggests that the virus may exploit these host proteins as binding receptors to infect adjacent cells [53]. In addition, several glycoproteins and tegument proteins have been reported to play a role in CCS. For example, the glycoproteins E and I (gE and gI, respectively) promote virion delivery [79]. Deletion of amino acids 167–244 in pUL51 or ablation of pUL7 expression hinder the concentration of gE at the junctional surfaces of Vero cells [80]. In the absence of gC, progeny virions bind more tightly to infected cells, indicating that gC facilitates virion detachment from infected cell surfaces. Consequently, gC also enhances the release of cell-free progeny virions at the end of the infectious cycle [83]. Similarly, glycoproteins K, M, and N (gK, gM, and gN) and tegument proteins UL11, UL16, and VP22 also participate in the transmission mechanism [20, 79]. In contrast, in CFR, the progeny traverse the plasma membrane via host-directed pathways involving Rab6a, Rab8a, and Rab11 and are then expelled into the extracellular environment through exocytosis [79]. Although certain viral and cellular factors have been implicated in CFR, the potential mechanisms remain poorly understood. One such viral factor is ICP27, as evidenced by the decreased CFR observed in ICP27 mutants [84]. Other factors, such as gC and host protein tyrosine phosphatase, are important for the spread of HSV-1 CFR [81]. ICP27-gC forms a regulatory axis that induces CFR specifically in tissues linked to reactivation, drawing parallels from the behavior of human cytomegalovirus, varicella-zoster virus, and Marek’s disease virus [79]. Furthermore, CFR and CCS engage in significant competition during vesicular formation and trafficking, as evidenced by the unusually high levels of CFR exhibited by the CCS-deficient gE mutant [80].

Recently, HSV-1 was reported to employ extracellular vesicles (EVs) for packaging and delivering viral components or infectious virions, significantly enhancing its transmission efficiency [85, 86]. HSV-1 hijacks the cellular vesicular secretion system and promotes EV secretion from infected cells. Previous studies have revealed that non-infectious EVs secreted by HSV-1-infected cells possess antiviral effects against HSV-1 due to their containment of host-restrictive factors, including STING, CD63, and Sp100 [87–89]. However, a recent study showed that Oct-1, a nuclear-localized transcription factor that initiates genome transcription, is packaged in non-virion-containing EV and exported from HSV-1- infected cells, which is then immediately transported into the nucleus of recipient cells to promote the subsequent round of HSV-1 infection [90]. EV-associated Oct-1 can enhance viral dissemination, and underline the heterogeneous nature and complexity of these non-infectious double-lipid particles in the HSV life cycle.

Determining how these cellular and viral factors modulate the CCS and CFR is important. Blocking the spread of viruses between cells and hosts by targeting CCS or CFR can greatly reduce viral transmission, alleviate HSV diseases, and prevent serious inflammatory complications and clinical and subclinical symptoms. The efficient transmission of HSV is of paramount importance for its utilization as an oncolytic virus in tumor therapy and as a tracer.

### Latent infection

Although most viruses are cleared by immune responses induced by lytic infection, a portion can escape host immunity, and access the nucleus of sensory neurons, and establish latent infection. HSV-1 commonly resides latently in the trigeminal ganglion (TG), whereas HSV-2 remains latent in the dorsal root ganglia (DRG). Currently, the molecular mechanisms of HSV latent infection are not clear, but three urgent issues need to be understood, as outlined below, to inform the development of antiviral drugs or vaccines.

First, what causes HSV to undergo latent infection? However, this topic remains largely unexplored. Available data suggest that the choice between latent and lytic infections is a stochastic process spanning several days rather than an instant decision upon HSV-1 entry into the TG from the cornea. The initial step towards establishing a latent infection, which is silencing, occurs very early, possibly during retrograde transport to neurons. Latency-associated transcripts (LATs) and microRNAs (miRNAs) are key factors in establishing latency and accumulate over a prolonged period [68]. In essence, the decision to develop a latent infection occurs early and is regulated by a combination of various factors rather than a singular event.

**Fig. 3:**
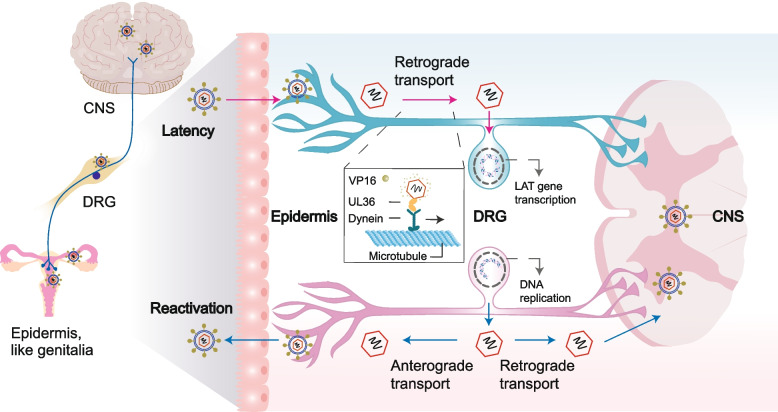
Establishment of HSV latency and reactivation in neurons. After primary infection in the epidermis, HSV enters sensory nerves innervating the skin or mucosa, undergoes retrograde axonal transport to reach the trigeminal ganglion, where it establishes a lifelong latent infection. The capsid containing pUL36 and other inner tegument proteins travels to the nucleus independently from tegument proteins like VP16. This process, together with histones and histone-modifying enzymes within nucleus, leads to DNA silencing. Conversely, the LAT gene sustains transcription and accumulation. Upon reactivation, HSV replicates and travels either via anterograde axonal transport to the peripheral epidermis, causing recurrent herpes, or retrograde axonal transport to the central nervous system, potentially leading to encephalitis or maintaining latency. CNS, central nervous system; DRG, dorsal root ganglia

Second, the mechanism by which HSV invades neurons remains unclear. There are several assumptions regarding neuron entry. It is widely accepted that the axon terminus near the peripheral epithelial cells is the initial site of HSV-1 attachment in neurons (Fig. 3) [91]. However, some studies have suggested that HSV-1 directly enters the cell body via membrane fusion [61]. Other studies suggest that HSV-1 enters neurons through a pH-independent fusion process between its envelope and the neuronal plasma membrane [92]. In contrast, studies have shown that nectin-1 is the main receptor in neurons, facilitating receptor-dependent membrane fusion and entry into epithelial cells [93, 94].

As the virus and tegument proteins enter the neurons, the outer tegument proteins dissociate into the cytoplasm, whereas the inner tegument proteins (VP1/2, UL36, and Us3) travel together with the nucleocapsids along nerve fibers to the nucleus of sensory neurons by interacting with the actin cytoskeleton (Fig. 3) [92]. Now, a pivotal question arises: What mechanism does the HSV rely on for its movement? Pegg found that the pUL36 protein can “hijack” dyneins and kinesins, causing them to leave epithelial cells and enter neural cells, thereby enabling HSV-1 transport from the cytoplasm to the nucleus along the meridian axis [95]. Rickard and Sollars demonstrated that pUL37 is also essential for the virus to move along nerve fibers, and mutated viruses with modified pUL37 region 2 (R2) cannot penetrate deep into the nervous system, but instead become stuck at the end of the nerve [96].

Based on these results, there is a new idea for the development of HSV vaccines against latent infections. The effectiveness of an R2-modified HSV-1 live virus vaccine against HSV-2 infection in guinea pigs was evaluated, exhibiting remarkable superiority. The release time of HSV was shortened from 29 days to approximately 13 days, and HSV-2 was undetectable in the neurons. Simultaneously, the neutralizing antibody level was three-fold higher than that of the other candidate vaccines [96]. The R2 vaccine can block HSV entry into the nervous system, thereby avoiding latent infection and neurological complications, which has significant implications for future vaccine development efforts.

Lastly, how does HSV establish and sustain a latent infection? The nature of latent infection is to maintain viral DNA silencing and block the expression of a large number of genes. HSV DNA is known to be silenced in heterochromatin during latent infection, and according to current reports, the reasons may be related to the following aspects. First, owing to the absence of HCF1 and VP16 in the neuronal nucleus, viral DNA undergoes gradual silencing by histones and histone-modifying enzymes within the nucleus, leading to heterochromatin. Some studies have indicated that HCF1 and VP16 are retained in the axons or cytoplasm, preventing their translocation to the neuronal nucleus to overcome heterochromatin and initiate transcription. Another hypothesis is that the distinctive neuronal architecture leads to the inefficient axonal transport of virion-associated regulatory factors [97–100]. Second, host immunity plays an important role in establishing and maintaining latency. Knipe and Sodroski discovered that interferon-inducible protein 16 (IFI16) restricts gene expression and replication of a nuclear DNA virus by maintaining or preventing the removal of repressive heterochromatin [101]. This study defines the impact of nuclear interferon-stimulated genes (ISGs) and provides the foundation for future antiviral strategies related to nuclear epigenetic silencing [101]. Additionally, the reactivation rate of latent infection in the central nervous system (CNS) is relatively low, possibly because of the robust immune response against HSV, which is exacerbated by the immune surveillance of microglia and astrocytes, which express a range of toll-like receptors (TLRs) [102]. Moreover, HSV latency in the human TG is related to T-cell accumulation, specifically the persistence of CD8+ T cells in the ganglia, which is likely triggered by parenchymal cells [103]. Third, there is a consensus that a neuron-specific promoter in the viral genome drives the expression of LATs, which play a major role in enhancing latency reactivation because viruses lacking LAT reduce latency and reactivation [104]. During latency, LAT produces multiple miRNAs, and two small non-coding RNAs (sncRNAs) [71, 105]. These miRNAs can interfere with viral and cellular gene expression, and several HSV-1 miRNAs can suppress the expression of key lytic regulatory factors, such as ICP0, ICP4, and ICP34.5 [106]. This suggests that miRNAs stabilize latency by mitigating the cytotoxic effects of spurious viral protein expression levels [107, 108]. miRNAs are also likely to target multiple host mRNAs, thereby altering the neuronal environment or suppressing antiviral responses. For example, MiR-138 can simultaneously regulate ICP0, Oct-1, and FOXC1 in the host to inhibit the expression of viral lytic genes, thereby creating favorable conditions for HSV latency [89]. Neuronal MiR-9 can facilitate HSV epigenetic silencing and latency by suppressing *Oct-1* and *Onecut* genes, as the nonspecific binding of Onecut with viral genes effectively decreases viral heterochromatin and increases the accessibility of viral chromatin [109]. The sncRNAs not only have anti-apoptotic activity but also induce HVEM overexpression by activating its promoter [54, 110]. HVEM plays a role in HSV latency and reactivation by controlling apoptosis and T cell activation and independently binding to gD [111, 112]. Overall, these results suggest that LAT helps establish and maintain HSV's long-term latent infection in the host by preventing neuronal apoptosis and suppressing host immune responses [113]. Nevertheless, some studies have found that LAT-mutant viruses can promptly achieve latency, albeit with a reduced number of neurons overall and fewer neurons harboring the virus, indicating that LAT may not be the sole determinant of latency or neuronal survival [114, 115]. Finally, HSV may express non-coding RNAs such as LATs and miRNAs, which are the only abundant viral gene products during latent infection. However, one study has reported that ICP0 is expressed and regulates viral chromatin to optimize latent infection during the establishment and maintenance of latent infection in mice [116]. Given its dual role, ICP0 may be a viable target for antivirals designed to combat both lytic and latent infections.

Although numerous hypotheses exist regarding the establishment and maintenance of latent HSV infections, the molecular mechanisms underlying neuronal latency remain unclear. One such controversial topic is whether neurons serve as immunologically privileged sites for HSV, enabling the virus to evade host antiviral responses. The latency of HSV is a complex phenomenon, either active or passive, resulting from either the host immune system successfully suppressing the virus from reaching nerve cells, or the virus actively entering a resting state within these cells to evade antiviral immunity. Furthermore, the nature of latency remains unclear, whether instantaneous or continuous, involving questions about whether low-level virus production persists and whether accumulated LAT and other products can disseminate to neighboring cells, thereby retaining infectivity. These questions can only be answered by relying on research. Hopefully, the mechanisms can be fully understood, and breakthroughs can be found to block HSV latency infection in neurons or virus recurrence.

### Reactivation

Sporadic reactivation events of latent infections occur within the host’s lifetime, particularly in immunodeficient individuals. These reactivation events exhibit remarkable heterogeneity, even within a single individual, in terms of position, intensity, and duration. Typically, the progeny virus travels to the site of primary infection or the neural system, potentially leading to viral shedding and transmission to other tissues or hosts. In some cases, it can initiate productive infections in the brain, resulting in encephalitis if they find their way to the CNS (Fig. 3) [54, 79, 117–119]. Currently, the mechanisms by which host and viral factors affect the HSV latent-lytic switch are partially understood. The nature of HSV reactivation involves extensive chromatin reorganization modulated by various factors to ensure adequate levels of viral gene expression for replication. Additionally, the virus must overcome antagonistic host responses to successfully transition from a latent to an active state. The broadly accepted view is that reactivation events are triggered by diverse stress signaling pathways, which can alter the silencing of histone modifications and initiate the transcription of viral genes [120, 121].

However, what are the stresses, and how do stress-signaling pathways trigger HSV reactivation? First, stress (acute, episodic acute, or chronic), fever, UV light, and heat stress can elevate the frequency of reactivation in humans by activating the glucocorticoid receptor (GR) [122]. GR exerts anti-inflammatory and immunosuppressive effects by inhibiting the transcriptional activity of protein 1 and nuclear factor kappa-light-chain-enhancer of activated B cells (NF-κB). This mitigates T cell activation, limits T helper subsets expansion, reduces T-cell co-stimulation, dampens innate immune and pro-inflammatory responses, and ultimately hinders the spread of viruses to peripheral cells and tissues [123–125]. Additionally, approximately 50% of TG sensory neurons express the GR, indicating that GR activation can directly induce reactivation from latency by stimulating viral gene expression [122, 126]. Second, the GR and specific stress-induced cellular transcription factors stimulate viral promoters, driving the expression of key viral transcriptional regulators such as ICP0, ICP4, ICP27, and VP16 [122]. ICP0 is a protein normally involved in reactivation and is rarely expressed during latent infection. Once latent infection is broken and reactivation begins, ICP0 performs epigenetic regulation of the viral genome and promotes viral replication. ICP0 inhibits the host immune defense and interacts with the Ub pathway to foster an environment conducive to lytic infection and reactivation of viral genomes from latency, relying on its RING finger E3 ubiquitin ligase activity [127]. ICP0 also disrupts ND10 structure and dissociates HDAC 1 and 2 from CoREST/REST to relieve repression, as mentioned in “Genome expression” [128, 129]. VP16 binds to the promoters of IE genes to initiate transcription, and concurrently, ICP0 and ICP4 are expressed as IE proteins, further stimulating the expression of E and L genes [68, 122, 130]. Third, HSV reactivation is caused by the loss of trophic support and the need to find a new foothold. Many cell types secrete nerve growth factor (NGF), the first identified neurotrophic factor that aids in neurite outgrowth, thereby facilitating HSV transmission and infection [131–133]. HSV-1 reduces the repulsive effect of epithelial cells on neurite outgrowth, particularly in the presence of NGF, whereas HSV-2 gG increases neurite outgrowth, thereby facilitating the spread of HSV to neurons [133–135]. Studies have demonstrated that applying anti-NGF antibodies to the eyes of rabbits with latent infection results in viral shedding, which aligns with increased reactivation [136]. Finally, host signaling pathways are vital in reactivation. Notably, during a de novo infection, cellular stress conditions exert a prolonged impact on neurons or the viral genome, effectively enhancing the reactivation potential. As an example, the c-Jun signaling pathway commences its regulatory function during the initial HSV-1 infection. It not only boosts the reactivation capacity by adjusting latency but also directly propels HSV-1 towards a fully reactivated state. However, the precise mechanism behind how the viral genome or neurons retain a memory of prior cellular stresses remains unclear [137, 138]. Furthermore, single-cell sequencing (scRNA‐seq) of reactivated neurons reveals that DNA damage–inducible 45 beta (Gadd45b), a host stress sensor, functions as a novel neural determinant for HSV-1 reactivation. Its subcellular localization correlates with ICP 4 expression, potentially serving as a predictive marker for successful reactivation [139].

Another issue worth discussing is the factors influencing the heterogeneity of HSV reactivation events. The prevailing assumption is that the degree of reactivation is positively correlated with the number of viruses invading during lytic infection and the quantity of latent viral DNA in nerve cells, which may be due to the high latency and relatively high reactivation potential and proportion [97, 140]. However, several studies have implicated host immune cells in playing a significant role in the reactivation of HSV-2 within the epithelium [141]. For example, HSV-2-specific tissue-resident memory CD8+ T cells (TRMs), a subset of CD8+T cells, are crucial for controlling viral reactivation and act as rapid responders to prevent reinfection or reactivation [142–144]. However, TRMs are not uniformly distributed in tissues, but rather clustered in hetero-dispersed aggregates at the dermal-epidermal boundary. Occasionally, they may not be well positioned to promptly contain the emergence of HSV-2, allowing latent viruses in different locations to evade immune control with varying probabilities, leading to heterogeneity [145]. The strength and duration of epithelial reactivation events are primarily determined by the temporal and spatial limitations placed on TRMs in containing each reactivation [79]. Moreover, the concentration of CD8+ T cells in the genital mucosa reliably predicts the duration and severity of viral reactivation, consistent with the recurrence tendency in immunodeficient individuals [141].

For every HSV genome that generates an infectious progeny, a greater number will likely recur but fail at some point. As viruses strive to overcome these reactivation obstacles, viral activity increases, and the likelihood of a robust counter-response from the host rises and progresses to each successive stage. The competition between the virus and the host revolves around maximizing viral production while carefully gauging the host’s capacity to support critical processes, such as DNA replication, virion synthesis, or dissemination to epithelial cells, to prevent the elimination of infected neurons without producing new viruses. These studies have identified some cellular factors, viral regulatory proteins, and signaling pathways that regulate reactivation, and further research is imperative to delve deeper into these mechanisms. Such explorations may reveal potential drug targets or novel therapeutic strategies aimed at reducing the frequency of reactivation due to latency and potentially new application opportunities.

## Host immune responses and virus immune evasion

Upon infection, innate immune responses are induced by viral antigens, serving as the first line of defense against HSV while fostering the emergence of adaptive humoral and cellular responses for long-term immunity. Nevertheless, certain viruses skillfully evade antiviral immune responses, invading and replicating successfully and establishing latency and reactivation [141]. HSV constantly competes for host immunity, apparently winning because the virus has successfully established a complete lifecycle. Consequently, exploring HSV immune evasion strategies holds immense significance, and it is worth comprehending the intricate immune interplay between HSV and the host, paving the way for the development of an effective vaccine capable of eliciting a robust immune response.

### Innate immunity

**Fig. 4:**
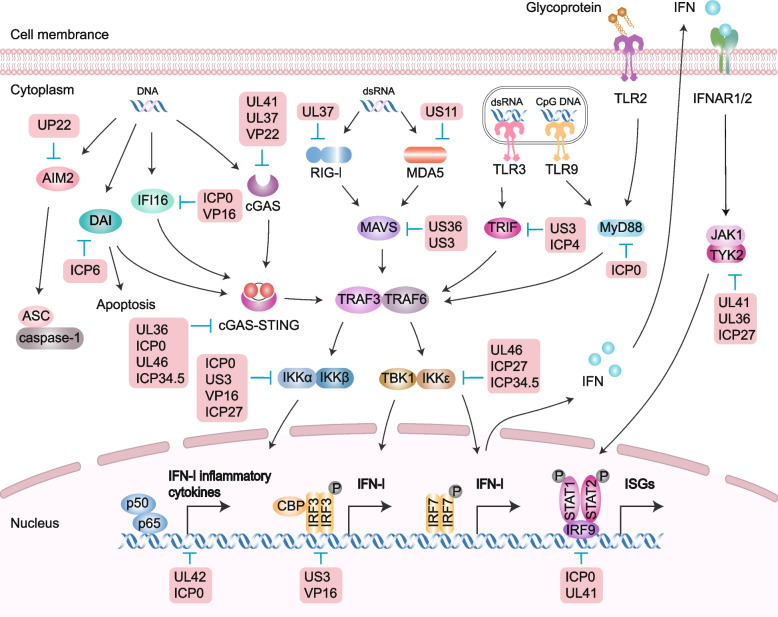
The host innate immunity and immune evasion mediated by HSV. TLRs, located at both the plasma membrane and endosomes, sense different ligands such as viral dsRNA, dsDNA, and glycoproteins. RLRs such as RIG-I and MDA5 detect RNA structures, and the cGAS-STING pathway senses dsDNA. They all activate IRF3 or NF-κB, inducing IFN-I and inflammatory cytokines. IFN-I stimulates ISG expression via the JAK-STAT pathway to limit HSV. NLRs such as AIM2 and DAI recognize dsDNA and lead to apoptosis and autophagy. The IFN-I, inflammatory cytokines, cellular responses and ISGs are induced for antiviral immunity, but HSV proteins highlighted in the red box can hijack multiple downstream steps of these signaling pathways, effectively suppressing these immune reactions. CBP, CREB-binding protein; P, phosphate

The innate immune system induces the initial response to HSV infection [15]. Host cells sense invading viruses via cellular pattern recognition receptors (PRRs) to elicit antiviral innate immune defense. PRRs can be generally categorized into several distinct families, including TLRs, RIG-like receptors, NOD-like receptors, C-type lectin receptors (CLRs), AIM2-like receptors, and cyclic GMP-AMP synthase (cGAS). These PRRs recognize a wide range of pathogen-associated molecular patterns derived from bacteria, viruses, fungi, and protozoa, as well as danger-associated molecular patterns. PRRs can recognize nucleic acids. Among them are DNA sensors, such as endosomal TLR9, cytosolic absent in melanoma 2 (AIM2), IFI16, DNA-dependent activator of interferon-regulatory factors (DAI), and cGAS. RNA sensors include TLR3, TLR7, TLR8, cytosolic retinoic acid-inducible gene I(RIG-I), melanoma differentiation-associated protein 5, NLR family pyrin domain containing 3, and nucleotide-binding oligomerization domain-containing protein 2. TLR2 recognizes viral glycoproteins [146]. Once these antigens are sensed, PRRs activate their adaptors, downstream interferon regulatory factors (IRFs), and NF-κB, leading to the expression of cytokines, chemokines, major histocompatibility complex (MHC), and co-stimulatory molecules to interfere with viral replication. Notably, type I interferons (IFN-α and IFN-β), a subgroup of cytokines, induce the expression of multiple ISGs, thereby creating an antiviral state in infected and surrounding cells, which blocks viral infection and limits its transmission, leading to an antiviral response by guiding IFN-responsive genes on adjacent cells to bind to the IFNα/β receptor and activate the JAK-STAT pathway to inhibit viral replication [101, 147, 148]. In addition, PRRs can trigger signal transduction and induce cellular processes, such as phagocytosis, autophagy, cell death, and inflammasome activation. These processes collaborate with the innate immune response to create a comprehensive network of antiviral host defense mechanisms (Fig. 4) [146].

However, to ensure long-term survival and generate significant progeny from infected cells, HSV employs diverse countermeasures, encompassing transcription shutoff, protein degradation, interaction competition, and enzymatic activity disruption, all of which involve multiple viral proteins, particularly IE proteins, tegument proteins, and other functional proteins, to evade host antiviral responses (Fig. 4) [52]. For instance, in the STING pathway, the HSV-1 UL37 tegument protein targets cGAS by deamidating an asparagine residue, thereby limiting the synthesis of cGAMP. Additionally, UL41 degrades cGAS via RNase activity, effectively evading the cGAS/STING-mediated DNA-sensing pathway [149, 150]. Furthermore, the downstream events of the cGAS-STING and DAI-STING are shared, which can be inhibited by the serine protease activity of HSV-1 UL24 to impair NF-κB activation [151]. In the TRAF3-TBK1-IRF3 pathway, UL36, a ubiquitin-specific protease, deubiquitinates TRAF3, thereby hindering stimuli-induced IRF3 dimerization and nuclear translocation, ultimately inhibiting IFN-β transcription [152]. Moreover, VP24 targets TBK1, hampering IRF3 phosphorylation, leading to the impairment of IFN-I generation and subsequent repression of ISGs in infected and nearby cells [153, 154]. As for RIG-I, the viral kinase Us3 specifically phosphorylates RIG-I, effectively blocking downstream signaling [155]. In addition, UL37 directly blocks the function of RIG-I by deamidating its helicase domain, a crucial component for sensing dsRNA products [156]. Myeloid differentiation primary response 88 (MyD88) is triggered by both TLR2 and TLR9, leading to the activation of NF-κB and type-1 IFN. ICP0, independently of other viral factors, effectively blocks the downstream signaling of MyD88 and reduces the levels of both MyD88 and its adaptor-like protein (Mal) through its E3 Ub ligase activity and cellular proteasomes [157, 158]. Conversely, ICP27 modulates the STAT-1 pathway by disrupting STAT-1 phosphorylation and nuclear accumulation [159]. Additionally, HSV-1 suppresses the activity of antiviral restriction factors by manipulating peptidylarginine deiminases (PADs). For example, HSV-1 infection enhances the citrullination of IFIT1 and IFIT2, which are induced by IFN and play crucial roles in antiviral and immunomodulatory responses [160]. In contrast, HSV interferes with cellular responses, as exemplified by ICP34.5, which binds to Beclin-1 and inhibits Beclin-1-dependent autophagy, a crucial antiviral mechanism, particularly within the nervous system [161].

During HSV infection, PRRs dynamically detect a range of molecular patterns throughout the viral life cycle, including the DNA genome, transcription-derived RNA species, unmasked cellular RNA, proteins, and peptides. This triggers innate immune signaling. Strategies that interfere with these manipulations could lead to novel antiviral therapies, and immune modulatory-deficient HSV mutants offer promising candidates for vaccines and oncolytic viral strains, further emphasizing the translational value of basic research. Notably, while PRRs recognize various viral components to induce type-1 IFN and thereby limit viral infection, they may also contribute to exacerbated inflammatory responses, such as brain inflammation and corneal infectious blindness [162]. Therefore, managing appropriate levels of inflammation or immunity is a crucial consideration for the future development of strategies targeting HSV and its associated complications.

### Adaptive immunity

**Fig. 5:**
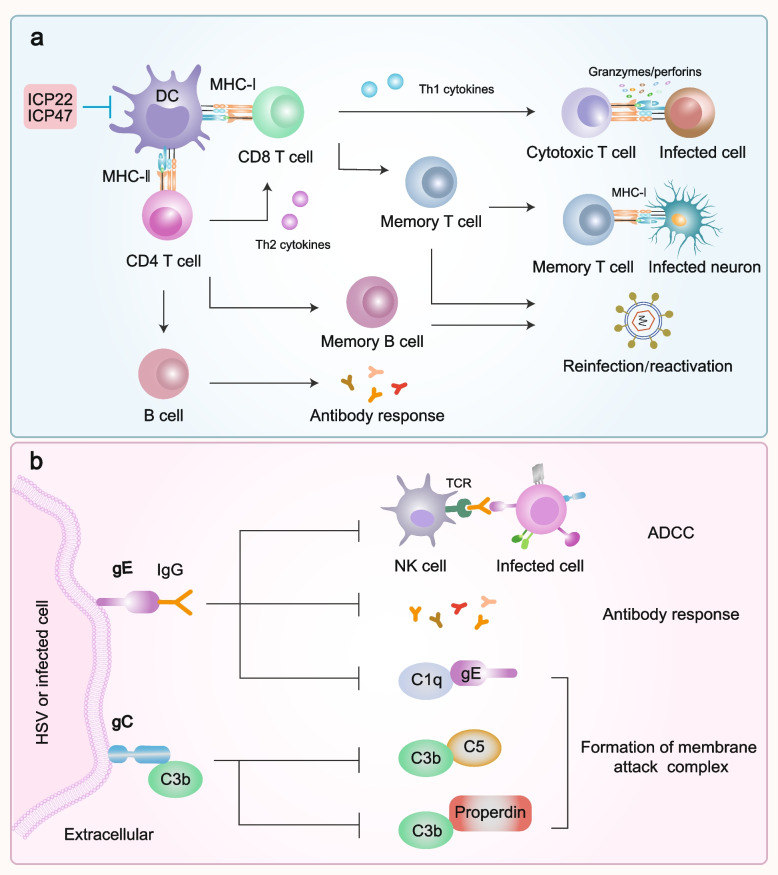
The host adaptive immunity and immune evasion mediated by HSV. **a** DC processes viral antigens and migrates to draining lymph nodes to present these antigens to activate CD8 T and CD4 T cells, thereby triggering both humoral and cellular responses. CD4 T cells produce Th1 cytokines that stimulate CD8 T cells, inducing CTL to eliminate infected cells, and Th2 cytokines can aid in the differentiation of B cells, leading to antibody production. Memory T cells continuously monitor HSV-infected neurons, and are ready to respond to reinfection. Meanwhile, memory B cells can produce a wide range of virus-specific antibodies, effectively limiting the potential for reinfection. However, HSV proteins such as ICP22 and ICP47, highlighted in the red box, can reduce MHC levels and inhibit the activation of T cells, thereby suppressing adaptive immunity. **b** gC can inhibit the complement system by binding to C3b. Since gE functions as a FcγR and binds with IgG, C1q cannot bind to gE, thus suppressing the complement system, and the virus-specific antibodies cannot recognize HSV and NK cells, resulting in suppression of antibody responses and ADCC. DC, dendritic cell; NK, natural killer cells; ADCC, antibody-dependent cell-mediated cytotoxicity

Unlike the innate immune system, adaptive immunity (humoral and cell-mediated responses) targets the pathogen and is more sophisticated, conferring enduring protection and playing a pivotal role during the early stages of infection, latent infection, and reactivation via CD4 and CD8 T cells in the genital tissue [163–165]. Upon HSV infection, DCs process viral antigens and migrate to the lymph nodes to present these antigens to activate T cells. During HSV infection, macrophages also contribute to the processing of HSV antigens and presentation to T cells. Moreover, the M1/M2 macrophage balance may influence HSV-induced cytokine production and eye disease in mice [166]. HSV-specific T cells are found in both active and healed lesions as well as in infected sensory human ganglia [121]. CD4+ T cells are mainly involved in primary infection, whereas CD8+ T cells contribute significantly to immune responses during latent genital herpes infection and recurrence, which is supported by the fact that the depletion of CD8+ T cells results in higher reactivation rates [167, 168]. Activated CD4+ T cells flow into the genital tissue in a CCR5-CCL5-dependent manner, peaking 1 week after infection. They orchestrate the anti-HSV adaptive immune response and assist B cells in antibody production. HSV-specific CD8+ T lymphocytes produce numerous cytolytic molecules that eliminate infected cells through cytotoxic T lymphocyte responses and release IFNs in response to viral antigens [121]. Although CD8+ T cell infiltration into vaginal tissue is limited under homeostatic conditions, TRMs respond quickly to HSV recurrence in an IFN-γ dependent manner, constituting the primary immune response in recurrent human infections [141, 166, 169, 170]. Moreover, MHC class 1 and T cell receptor engagement occur at the contact region between neurons and memory CD8+ T cells (Fig. 5a) [171]. However, CD8+ T cells did not respond to LATs. TRMs exhibit uneven distribution in tissues and, in certain instances, fail to contain the virus promptly [169, 170, 172–174]. Consequently, the virus can seize this opportunity to evade immune surveillance, leading to reactivation and transmission [145]. Moreover, studies of herpes keratitis and herpetic stromal keratitis have shown that Tregs play a beneficial role in minimizing viral immunological lesions. By inhibiting the proliferation of CD4 and CD8 T cells and suppressing the release of inflammatory cytokines and chemokines such as IL-2, IL-6, and CCL3, Tregs mitigate the generation, migration, and harmful effects of pathogenetic T cells on the cornea [166, 175].

In addition to the escape time window of the host immune system, HSV has evolved various immune escape mechanisms to avoid clearance and subsequent recurrence. For instance, HSV employs ICP47, which targets transporters linked to antigen processing in the endoplasmic reticulum. This leads to reduced antigen presentation on MHC-I molecules by DCs. ICP47 impedes antigen translocation to this organelle, preventing the loading of viral antigenic peptides onto MHC-I molecules [176]. HSV can also reduce the capacity of DCs to activate T-cells through ICP22, which binds to the CD80 promoter and downregulates the expression of the co-stimulatory molecules CD80 and CD86 on the cell surface [48, 177, 178]. Reports indicate that both HSV-1 and HSV-2 can inhibit autophagosome formation in DC, interfere with cellular degradation processes, affect antigen presentation to CD8+ T cells, and hinder DC migration from infected tissues to the corresponding lymph nodes, likely reducing the efficacy of DC in activating CD4+ and CD8+ T cells at this site (Fig. 5a) [48, 179–182]. In addition, HSV protects infected cells from natural killer (NK) cell-mediated apoptosis by inhibiting the release of cytotoxic molecules from NK cells [183, 184]. Overall, these findings support the theory that HSV has developed multiple mechanisms and strategies to undermine the functions of DCs, NK cells, and T cells and potentially negatively affect host adaptive immune responses [154].

Another important immune escape mechanism is the restriction of the immune response by HSV gC and gE. gC can bind to complement component C3b, thereby blocking complement component C5 and properdin to activate the alternative and classical complement signaling pathways, respectively. Consequently, this mechanism prevents the formation of a membrane attack complex that lyses infected cells and impairs the ability of B and T cells to enhance immunity [185–188]. In addition, the Fc domain of gE functions as an IgG Fc receiver (FcγR), which binds to IgG and blocks antibody-dependent cell- mediated cytotoxicity and C1q binding [33, 189]. Notably, the FcγR of gE can block antibodies against HSV and many other viruses, exerting a broad inhibitory effect on all immune pathways associated with Fc, thus elevating the risk of infection with various pathogens and predisposing to immune-related diseases (Fig. 5b). However, it is interesting to note that the vaccine targeting gC2/gD2/gE2 has achieved phased success, with gD2 blocking viral entry and gC2 and gE2 blocking immune evasion, suggesting its outstanding potential in the fight against HSV infection [32].

In summary, although HSV invasion can induce host innate and adaptive immunity and cellular responses to eliminate or restrict HSV replication and transmission, sophisticated countermeasures have been devised to neutralize these anti-immune responses, thereby ensuring long-term survival and replication. Consequently, continually refining and consolidating our comprehension of these mechanisms to enhance our understanding of HSV-host interactions in immune responses is imperative. This will pave the way for the development of innovative antiviral strategies, vaccines, and oncolytic viruses.

## Progress in HSV vaccine development

As described above, the biological complexities, pathogenesis, and immune evasion mechanisms of HSV infection are profound, leading to complex clinical symptoms and posing significant challenges to the development of antiviral drugs and vaccines. The FDA has not approved genital herpes vaccines despite 75 years of effort, including attenuated live, nucleic acid, inactivated, subunit, genetically engineered live virus, and synthetic peptide vaccines [190]. The majority of vaccine candidates have fallen short in clinical stage I/II or even preclinical research, with only a minority advancing to phase 3 trials, yet none have achieved the desired level of efficacy [50, 191]. With regard to the development of HSV vaccines, given our knowledge of the pathogenesis of the virus, an effective vaccine would likely stimulate innate and adaptive immunity, encompassing both humoral and cellular responses, which are mainly determined by antigens and adjuvants selected as immunogens. However, based on the outcomes of various vaccine candidates tested in animals and humans using diverse platforms, antigens, and adjuvants, the failed vaccines may have overlooked antigens crucial for eliciting and maintaining a robust immune response despite several antigen screening processes [15].

Although these candidate vaccines have only generated partial success, each vaccine offers unique advantages and disadvantages, which are summarized below and serve as a valuable reference for future vaccine development efforts (Table 2). HSV vaccines can be classified as prophylactic or therapeutic based on the characteristics of the viral infection. Prophylactic vaccines are aimed at healthy individuals and are designed to establish a defensive barrier on the skin and mucous membranes to prevent viral invasion. The primary objective was to target the initial HSV infection and prevent the formation of latent infections. A successful prophylactic genital herpes vaccine should accomplish the following: prevent both clinical disease and subclinical infection, reduce the risk of inadvertent transmission to non-vaccinated partners (both males and females), maintain durable immune protection against HSV invasion and latency, provide cross-protection against both HSV-1 and HSV-2 genital infections, and effectively prevent maternal and neonatal herpes infections following female immunization [190–192]. Conversely, therapeutic vaccines are intended for the population already infected with the virus, aiming to alleviate clinical symptoms, prevent disease progression, and even suppress herpes recurrences. Simultaneously, it should have long-term effectiveness for both sexes, provide cross-protection against both HSV-1 and HSV-2 and be suitable for individuals of all ages.

**Table 2:** The development of HSV vaccine candidates

Candidate type	Candidate	Developer	Antigen&adjuvant	Status	Start year	Identifier	Classification	Population
Subunit vaccine	GSK208141	GSK	gD	Phase 1 completed	1992	NCT00698893	Prophylactic	16 male and female
				Phase 2 completed	1992	NCT00697567		80 male and female
				Phase 3 completed	2003	NCT00224471		671 female
			gD2	Phase 2 completed	1995	NCT00698490		130 male and female
			gD2t&MPL	Phase 3 completed	1996	NCT00699764		2491 male and female
				Phase 3 completed	1996	NCT00698568		7460 male and female
			Simplirix: gD&alum and dPML	Phase 3 completed	2003	NCT00057330		8323 female
			gD2&AS04	Phase 3 completed	2004	NCT00224484		5960 female
	GSK3943104A	GSK	—	Phase 1 Phase 2 recruitment	2022	NCT05298254	Therapeutic	332 male and female
	GEN-003	Genocea	gD2△TMR, ICP4.2&Matrix M2	Phase 2 completed	2012	NCT01667341	Therapeutic	143 male and female
				Phase 2 completed	2014	NCT02114060		310 male and female
				—	2015	NCT02300142		37 male and female
				Phase 2 completed	2015	NCT02515175		131 male and female
				Phase 2 terminated	2017	NCT03146403		33 male and female
	gB2/gD2	Novartis	gB2/gD2 & MF59	Phase 3 completed	1999	—	Prophylactic	2393 male and female
	gC2/gD2/gE2	BioNTech	gC2, gD2 and gE2&CpG and alum	—	2017	—	Prophylactic	—
	G103	Sanofi	gD2, UL19 and UL25&GLA-SE	Phase 1 stopped	2020	NCT04222985	Therapeutic	24 male and female
	—	MedImmune	gB2, gD2 and UL40&CpG	—	—	—	Prophylactic	—
	NE01-gD2/gB2	—	gB2 and gD2 in nanoemulsion adjuvant	—	—	—	Prophylactic/therapeutic	—
Synthetic peptide vaccine	HerpV	Agenus	Peptides&QS-21	Phase 2 completed	2012	NCT01687595	Prophylactic	80 male and female
DNA vaccine	pPJV7630	PowderMed	Ubiquitinated and unmodified gD2	Phase I completed	2006	NCT00310271	Therapeutic	42 male and female
	VCL HM01	Vical	—	Phase 1 Phase 2 completed	2013	NCT02030301	Therapeutic	165 male and female
	VCL-HB01	Vical	gD2+/UL46 and Vaxfectin	Phase 2 completed	2016	NCT02837575	Therapeutic	261 male and female
	COR-1	Anteris	gD2 codon optimized	Stopped after Phase I/IIa trial	2016	—	Therapeutic	40 male and female
mRNA vaccine	GSK-4108771A	GSK	gE/gI (SAM)	Phase 1 terminated	2021	NCT04762511	Prophylactic	17 male and female
	BNT163	BioNTech	gC2/gD2/gE2	Phase 1 recruiting	2022	NCT05432583	Prophylactic	248 male and female
	mRNA-1608	Moderna	—	Phase 1 Phase 2 actived	2023	NCT06033261	Therapeutic	365 male and female
Live attenuated and replication defective vaccine	HSV15	Sanofi	UL5, UL29 deletion	Phase 2 terminated	2020	NCT04222985	Therapeutic	24 male and female
	HSV529	Sanofi	UL5, UL29 deletion	Phase I completed	2013	NCT01915212	Prophylactic	69 male and female
				Phase I completed	2015	NCT02571166		10 male and female
	RVx-201	Rational	ICP0 deletion	—	—	—	Therapeutic	—
	RVx-2001	Rational	HSV-2	—	—	—	Prophylactic	—
	RVx-1001	Rational	HSV-1	—	—	—	Prophylactic	—
	Delta gD-2	X-Vax	gD deletion	—	—	—	Prophylactic	—
	ICP10△PK	AuRx	ICP10△PK	Stopped after Phase I/IIa	2002	—	Therapeutic	—

*dPML* 3-deacylated form of Monophosphoryl Lipid A; *gD2△TMR* a transmembrane deletion mutant of glycoprotein D; *CpG* cytosine-phosphate-guanine; *GLA-SE* glucopyranosyl lipid A formulated in a stable oil-in-water emulsion; *SAM* self-amplifying mRNA; —: representative information not disclosed. All information comes from https://classic.clinicaltrials.gov

Currently, subunit vaccines targeting viral glycoproteins are being extensively studied. These glycoproteins, especially gD, block cell entry, followed by gB, gC, and gE, and are often combined with various adjuvants to enhance immunity and prevent host cell entry and cell-to-cell transmission [193–196]. Unfortunately, some vaccines that have been proven to be effective in animal models have recently been discontinued owing to their failure in human clinical trials. A study sponsored by GlaxoSmithKline revealed that the gD2 vaccine, administered with MPL and alum as adjuvants, showed a high efficacy of 74% in HSV-1/HSV-2 seronegative women but was not efficacious in HSV-1 seropositive and HSV-2 seronegative women. It was not effective in men regardless of their serological status, raising concerns about sex differences in vaccine efficacy [191, 197]. To further assess its efficacy in women, the Simplirix vaccine was evaluated in a group of young women aged 18–30 years who were seronegative for HSV-1 and HSV-2. The vaccine exhibited efficacies of 58% against HSV-1 and 20% against HSV-2 [198]. The reason this finding differs from the previous studies may be the difference in the studied populations, as the attack rates of HSV-2 genital disease in prior studies were high among uninfected women in discordant couples and were significantly reduced by the vaccine. Regarding the gD2 vaccine, significant protection against HSV-1 infection, but not HSV-2, may be due to low-dose infection of HSV-1, as gD1 and gD2 amino acids share 89% homology [198]. The gD2 and gB2 subunit vaccines adjuvanted with MF59 can induce high levels of HSV-2 specific neutralizing antibodies in HSV-2 seronegative discordant couples and HSV-2 seronegative couples at sexually transmitted disease clinics; however, the overall efficacy for preventing HSV-2 infection was only 9% [199]. Although gD can elicit human immune responses and extensive research has been conducted on vaccines incorporating gD along with diverse adjuvants and platforms, these efforts have yielded only partial success. The limited effectiveness of existing vaccines targeting gD and/or gB can be attributed to the lack of a potent antigen [200]. HSV encodes a plethora of genes capable of immune evasion, which complicates vaccine development.

Recently, a trivalent subunit vaccine of gD2/gC2/gE2 administered with CpG and alum as adjuvants showed significant immune responses and protection in rhesus macaques and female guinea pigs, in which gD2 blocked viral entry, whereas gC2 and gE2 suppressed immune evasion [32]. In rhesus macaques, vaccine-induced plasma and mucosal-neutralizing antibodies stimulated CD4+ T cell responses and exhibited a remarkable efficacy of 97% against HSV-2 [32]. Similarly, in guinea pigs, the efficacy against acute disease and recurrent genital lesions reached 97%, showing both preventive and therapeutic effects and preventing the shedding of replication-competent viruses [32]. Combined with the unique advantages of mRNA vaccines, BNT163, a trivalent mRNA vaccine for gD2/gC2/gE2 encapsulated in lipid nanoparticles, was developed by BioNTech [201, 202]. BNT163 exhibited significant immunogenicity and efficacy in mice and guinea pigs. The efficacy against clinical and subclinical infections reached 63/64 and 8/10 in mice and guinea pigs, respectively, significantly reducing the risk of transmission to partners and newborns. Immunological assays showed that the trivalent mRNA vaccine was superior to the trivalent proteins in stimulating serum and vaginal IgG antibodies, serum neutralizing antibodies, and antibodies targeting crucial gD2 epitopes involved in entry and cell-to-cell spread, CD4+ T cell responses, and T follicular helper and germinal center B cell responses in mice [203]. Second, BNT163 showed long-term protection in guinea pigs and mice, lasting approximately 8 months and 1 year, respectively. This durable protection is likely attributable to the generation of high neutralizing titers and a robust B-cell immune memory that persists for up to a year [204]. Third, the mRNA vaccine also generated cross-reactive antibodies against vaginal HSV-1 infection and latent infection, resulting in complete protection from death and genital disease in all mice infected with HSV-1 (54/54, 100%) and HSV-2 (20/20, 100%) and prevented HSV DNA from reaching the dorsal root ganglia in a high proportion of mice infected with HSV-1 (29/30, 97%) and HSV-2 (10/10, 100%). Overall, BNT163 provides comprehensive protection against HSV-1 and HSV-2 genital herpes in animals, making it a promising candidate for further development [193, 205]. Finally, a study involving female mice immunized before mating and newborns infected intranasally with HSV-2 suggested that the efficacy of mRNA and protein vaccines in newborns was 117/120 and 154/160, respectively. Both vaccines induced comparable IgG binding and neutralizing antibody levels in mothers and newborns, successfully protecting first- and second-litter newborns from disseminated infections based on virus titers in multiple organs [206]. Collectively, these four aspects underscore the ability of trivalent mRNA vaccines to effectively prevent genital herpes. Based on animal models, BNT163 is currently the closest to an ideal vaccine, and a clinical trial is being conducted by BioNTech. The success of both trivalent subunit and mRNA vaccines further underscores the significance of multiple vaccine antigens, particularly those related to immune escape, for inducing and maintaining effective immune responses during the development of an HSV vaccine.

Other candidate HSV vaccines exist that have demonstrated partial success and require further investigation. One such vaccine, HerpV, is a recombinant human heat shock protein 70 polyvalent peptides complexed with 32 synthetic HSV-2 peptides, adjuvanted with QS-21. In a phase 1 trial, HerpV elicited HSV-2-specific CD4+ and CD8+ T cells and reduced HSV-2 shedding by 15% following initial vaccination [207, 208]. Another vaccine, GEN-003, comprises two recombinant T-cell antigens: an internal fragment of ICP and a transmembrane deletion mutant of gD2 with a matrix-M2 adjuvant and a saponin-based lipid particle. The vaccine significantly reduced viral shedding (approximately 40%) and lesion rates while stimulating humoral and cell-mediated antigen-specific immune responses. However, GEN-003 has been acquired by another company [209, 210]. Moderna predicts that mRNA-1608 can inhibit HSV-2 genital herpes and provide cross-protection against HSV-1, with clinical trials already underway. Another strategy is the live attenuated HSV vaccine, which specifically deletes genes to limit infection and replication while maintaining immunogenicity and induces extensive immune responses by supplying a wider range of antigens. The COR-1 vaccine, a gD2 codon-optimized DNA vaccine, demonstrated both cellular and humoral responses in murine models and reduced viral shedding in humans [209]. The VC2 vaccine, which contains HSV with partial deletions in the gK and UL20 genes, prevents HSV from entering the neuronal axons [211]. This vaccine reduces acute and recurrent HSV-2 disease, viral shedding, and the amount of virus detected in neurons [96, 212, 213]. However, modifying the virus may carry risks, such as reducing immunogenicity or enhancing virulence [96].

The ongoing clinical and preclinical vaccine efforts are dependent on the present comprehension of HSV biology and immunopathogenesis within host cells [214]. According to nearly 80 years of HSV vaccine development, most vaccines are primarily constrained by the identification of vaccine antigens capable of effectively inducing and sustaining robust immune responses, encompassing both humoral and cellular immunity [215]. The promise lies in subunit and mRNA vaccines, which provide a gateway to present the immune system with complex antigenic compositions, potentially including T cell and B cell epitopes. Furthermore, the combination of subunit vaccines with specific adjuvants and vaccine formats has introduced a novel approach for exploring future options in HSV vaccination. The next challenge concerns the vaccine development technology itself, particularly mRNA vaccines and LNP delivery systems. mRNA vaccines possess numerous unique advantages and have demonstrated superior efficacy compared to subunit vaccines, pointing to a promising pathway. Another obstacle is likely the absence of ideal animal models, as guinea pigs and mice currently used cannot comprehensively evaluate the effectiveness of HSV vaccines against lytic, latent, and reactivation infections. Therefore, constructing an ideal animal model is a critical priority for the future. Alongside these limitations, we also face additional challenges, including viral culture systems, injection methods, and adjuvant use, which indicate the direction of our future efforts.

## Application prospects of HSV

### Oncolytic virotherapy

Oncolytic viruses (OVs) are a promising emerging class of anticancer immunotherapies that use replication-competent viruses to specially target and lyse tumor cells. During this process, the virus and tumor antigens recruit more immune cells, boosting the elimination of residual tumor cells and enhancing immune cell infiltration to reshape the tumor microenvironment [216, 217]. To enhance the tumor selectivity of OVs, improve their replication efficiency, minimize pathogenicity, and bolster immunogenicity, they usually are genetically engineered through the deletion or modification of viral genes, modification of virus surface proteins, insertion of tumor specific promoters into the genome, or fusion of tumor cell specific antibodies [216, 218].

**Fig. 6:**
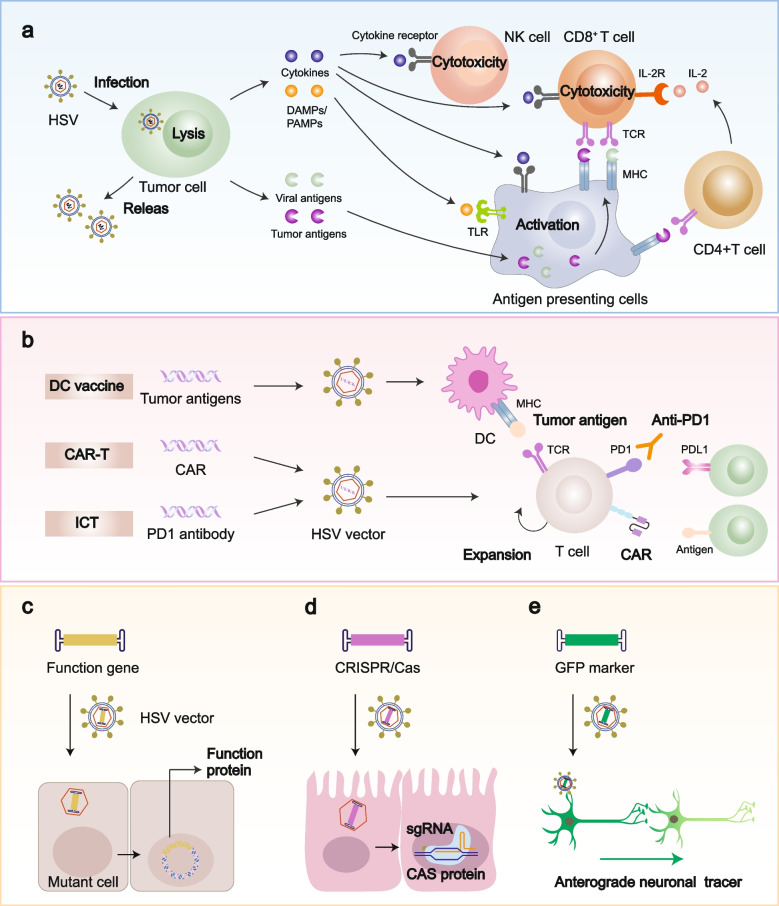
The applications of HSV. **a** HSV serves as an oncolytic virus to selectively infect, replicate, and lyse tumor cells. OV also eliminates distant and uninfected tumor cells via releasing progeny virions. Meanwhile, OV induces immune responses to amplify anti-tumor activity. The infected tumor cells release cytokines like type I IFNs, DAMPs, PAMPs, as well as viral and tumor antigens, which aid in maturing and recruiting antigen-presenting cells (APCs), inducing and activating tumor specific T cells and NK cells, to enhance immune responses. **b** HSV acts as a viral vector while also contributing to immune therapy. It introduces tumor antigens into DC as an anti-tumor vaccine, the DC also assists in the activation and expansion of tumor-specific cells to enhance immune responses. It also introduces CAR into T cells to generate CAR-T cells, enhancing the targeting of tumor cells. In addition, the expression of checkpoint inhibitors like PD1 antibodies by HSVs helps in blocking PDL1, leading to antitumor activity. **c** HSV acts as a viral vector carrying extra-functional genes to the mutant cell to restore its function by the expression of functional proteins. **d** HSV also carries gene editing systems like CRISPR/Cas to engineer genes, which can be used in the construction of disease models and anti-tumor therapy. **e** Due to its neurotropism, HSV can act as an imaging and tracing tool for neuroscience. It can be attached to a GFP anterograde transneuronal tracer. OVs, oncolytic viruses; IFNs, interferons; DAMPs, danger-associated molecular patterns; PAMPs, pathogen-associated molecular patterns; APC, antigen-presenting cells; NK, natural killer cells; DC, dendritic cell; CAR, chimeric antigen receptors; CAR-T, chimeric antigen receptor T-cell immunotherapy; ICT, immune checkpoint therapy

Currently, HSV is a widely used OV in clinical practice. It possesses several features that favor its use for oncolytic virotherapy (Fig. 6a). First, HSV-1 demonstrates potent infectivity, completing its replication cycle within 10 h and rapidly releasing a multitude of progeny viruses. This efficiency surpasses that of other common viruses, such as adenoviruses. Additionally, CCS promotes the efficient spread of these progeny viruses within tumors, resulting in effective tumor clearance or shrinkage. Second, HSV infection stimulates the secretion of cytokines such as granulocyte-macrophage colony-stimulating factor (GM-CSF), TNF-α, and IFN from tumor cells, thereby attracting more antigen-presenting cells (APCs) and activating T cells. As tumor cells lyse, viral and tumor antigens are released, further activating the host immune system. As a substantial influx of T cells is attracted to the tumor site, their infiltration within the tumor microenvironment is significantly strengthened, ultimately leading the transformation of “cold” tumors into “hot” ones. RNA sequencing (RNA-seq) has identified Visfatin in the responsive tumors following OV treatment. Visfatin promotes the antitumor efficacy of OV by remodeling the tumor microenvironment, specifically by enhancing CD8+ T cell and DC cell infiltration and activation, repolarizing macrophages towards the M1 like phenotype, and reducing Treg cell numbers [219]. This enhances the anti-tumor effects and treatment durability and provides immune-mediated tumor-killing therapy for patients unresponsive to inhibitor immunotherapy. Third, its ability to infect almost all cells, including immune and nerve cells, indicates its unique potential to induce strong immune responses and penetrate the blood-brain barrier, making it useful for treating neurological diseases. Studies have shown that oncolytic HSV-1 is effective against CNS cancers, such as glioblastoma, improving patient survival and quality of life. Finally, HSV-1 can effectively infect various experimental animals, making it highly suitable for preclinical in vivo studies [218, 220–222].

Recently, clinical trials have witnessed encouraging advancements in oncolytic viral therapies using HSV-1 (Table 3). T-VEC, the first OV product approved as a drug in Europe and the USA in 2015, is used to treat melanomas that cannot be completely removed surgically [220]. T-VEC is a second-generation oncolytic HSV-1 with deletions of ICP34.5 and ICP47 and the incorporation of human GM-CSF. These strategies bolster cancer-targeting replication, minimize virulence, hinder replication in healthy cells, and enhance antigen presentation and anti-tumor immunity. Studies have indicated that combining T-VEC with checkpoint inhibitors, such as Yervoy and Keytruda, gives a remarkable response rate of 62%, with most patients experiencing > 50% tumor reduction and exhibiting good tolerance and high efficacy. Furthermore, Teserpaturev/G47△ is a third-generation recombinant oncolytic HSV-1, with the deletion of ICP34.5 and ICP47 and the inactivation of ICP6. Owing to its high efficiency and specificity in humans, it was globally recognized as the first OV drug for glioblastoma treatment [221]. CAN-3110, an oncolytic herpes virus (oHSV), has demonstrated a significantly enhanced replication ability and glioblastoma treatment effectiveness in clinical trials [223, 224]. Furthermore, T3011, a recombinant oncolytic HSV-1 with insertion of IL-12 and PD1 and deletion of one copy of ICP34.5 to enhance anti-tumor activity and safety, was manufactured by ImmVira. Recent clinical data have suggested that intratumoral injection of MVR-T3011 (MVR-T3011 IT) combined with pembrolizumab is safe and tolerable and has the potential to modify the tumor microenvironment and overcome immune tolerance in malignant tumors. Additionally, BS006, a new oncolytic HSV equipped with bispecific antibody (BsAb) molecules targeting PDL1/CD3 (oHSV2-PDL1/CD3-BsAb) for human malignancies, has been approved for the initiation of phase 1 clinical trials. As a globally pioneering oncolytic viral drug, BS006 is expected to provide new treatment options for patients with cancer [225]. Other promising oHSV products, including VG161, OrienX010, and GM-GSF, are also in the pipeline [226–228].

**Table 3:** The development of oncolytic herpes virus (oHSV) candidates

Candidate	Gene engineering	Disease	Developer	Status	Identifier
Imlygic (T-VEC)	Delete ICP34.5 and ICP47, insert GM-CSF into HSV-1.	Melanoma	Amgen	Approved by FDA in 2015	
Delytact (G47△)	Delete ICP34.5 and ICP47, inactivate ICP6 in HSV-1.	Malignant gliomas	Daiichi sankyo	Approved by Japan in 2021	
G207	Delete ICP34.5, inactivate ICP6 in HSV-1.	Recurrent high-grade glioma	Gregory	Phase 2	NCT04482933
MVR-T3011	Express anti-PD1 antibody and IL1 in HSV-1.	Advanced colorectal cancer	ImmVira	Phase 1	NCT06200363
OrienX010	Express recombinant hGM-CSF in HSV-1.	Melanoma	OrienGene	Phase 1	NCT04197882
VG161	Express recombinant human IL12/15-PDL1B in HSV-1.	Advanced pancreatic cancer, metastatic gastric cancer	Virogin	Phase 1 Phase 2	NCT06124001
VG301	Express recombinant CD3-CEACAM6-BsAb in HSV-1.	CEACAM6-expressing tumors	Virogin	—	—
RP1	Express hGM-CSF/GALV-GP-R- in HSV-1	Melanoma	Replimune	Phase 1	NCT06216938
RP2	Express CTLA4 in HSV-1.	Solid tumor	Replimune	Phase 1	NCT04336241
TBI-1401 (HF10)	Delete UL56 in HSV-1.	Melanoma	TAKARA	Phase 2	NCT03153085
HSV1716	Delete ICP34.5 in HSV-1.	Malignant Pleural Mesothelioma	Virttu	Phase 1 Phase 2	NCT01721018
BS001	Delete ICP34.5 and ICP47, insert hGM-CSF in HSV-2.	Solid tumor	Binhui	Phase 1	NCT05954091
BS006	Express PD-L1/CD3-BsAb in HSV-2.	Solid tumor	Binhui	Phase 1	NCT05938296
ONCR-GBM	Express IL-12 and a PD1 antagonist nanobody in HSV-1.	Glioblastoma	Oncorus	—	—
R3616	Delete ICP34.5 in HSV-1.	Pancreatic cancer colon cancer	Genentech	—	—

*hGM-CSF* human granulocyte macrophage colony-stimulating factor; *FDA* Food and Drug Administration; —: representative information not disclosed. All information comes from https://classic.clinicaltrials.gov

### Gene and immune therapies

HSV has significant potential as a gene therapy vector owing to several key advantages. First, their strong genetic load capacity enables them to carry complex regulatory elements and a wide range of exogenous genes. Second, HSV demonstrates efficient transduction in various cell types. Third, its ability to evade host immunity facilitates repeated administration while minimizing immune toxicity. Finally, its lack of integration with the host genome ensures that it remains non-oncogenic [218, 220, 229, 230].

HSV vectors can correct defective genes by transmitting normal genes, thereby offering a potential therapeutic strategy for hereditary diseases (Fig. 6c). For instance, cystic fibrosis, a genetic disease caused by mutations in the *CFTR* gene, leads to dysfunctional or absent CFTR proteins and accumulation of mucus in the lungs, resulting in persistent lung infections and progressive lung deterioration. KB407, a modified HSV-1 vector, effectively carries two copies of the *CFTR* to respiratory cells in the lungs, providing a treatment option for all patients with cystic fibrosis, regardless of their specific genetic mutation. Similarly, dystrophic epidermolysis bullosa, a rare genetic blistering disease caused by mutations in *COL7A1*-the gene encoding type VII collagen (C7), leads to absent or dysfunctional anchoring fibrils and disrupts the adhesion of the epidermis to the dermis [231]. B-VEC, a replication-defective HSV-1 vector designed to restore functional C7 protein through the delivery of functional *COL7A1*, can treat wounds in patients 6 months or older with dystrophic epidermolysis bullosa caused by *COL7A1* mutations (Table 4) [39, 232, 233].

**Table 4:** The development of HSV in gene therapy candidates

Candidate	Gene engineering	Disease	Developer	Status	Identifier
Vyjuvek (B-VEC)	Express COL7A1 in HSV-1.	DEB	Krystal	Approved by FDA in 2023	
KB301	A non-integrating HSV-1 vector expressing COL3A1.	EB	Krystal	Phase 1	NCT04540900
KB105	A replication-incompetent HSV-1 expressing TGM1.	TGM1-deficient ARCI	Krystal	Phase 2	NCT05735158
KB407	A replication-defective HSV-1 expressing full length human CFTR.	CF	Krystal	phase 1	NCT05504837
BD111	Delivery of CRISPR/Cas9 mRNA targeting the HSV-1 genome.	HSK	BDgene	—	NCT04560790
WG1025	A replication-defective HSV-1 expressing COL7A1.	DEB	WellGene	—	—
Telaria	HSV-1	DEB	Replay	—	—

*DEB* dystrophic epidermolysis bullosa; *EB* epidermolysis bullosa; *TGM1* Transglutaminase 1; *ARCI* autosomal recessive congenital ichthyosis; *CF* Cystic Fibrosis; *HSK* HSV-1 Keratitis; *FDA* Food and Drug Administration; —: representative information not disclosed. All information comes from https://classic.clinicaltrials.gov

In addition to gene therapy for hereditary diseases, HSV vectors exhibit significant potential for immunotherapy (Fig. 6b). They can carry specific genes or drugs to directly infect target cells and release therapeutic substances, thereby enabling more comprehensive or personalized tumor treatment strategies. For instance, HSV can deliver immune-stimulating or anticancer genes to tumor cells, activate the immune system, induce apoptosis, and inhibit tumor cell proliferation. Furthermore, HSV can introduce chimeric antigen receptors (CARs) into T cells, enhancing their ability to recognize specific tumor antigens. Additionally, HSV vectors carrying genes that regulate the tumor microenvironment facilitate CAR T-cell targeting and tumor infiltration. The combination of HSV vectors and CAR-T cells has demonstrated promising therapeutic effects and offers new strategies for tumor immunotherapy. Furthermore, T cells express checkpoint inhibitor antibodies against HSV that block PDL1 or CTLA4, resulting in anti-tumor activity [222, 224, 234, 235].

Remarkably, HSV vectors can also transport nerve growth factors or other therapeutic proteins across the blood-brain barrier to the damaged nervous system, owing to their neurotropism. In autoimmune neurological diseases, such as multiple sclerosis, HSV carriers can effectively deliver anti-inflammatory factors or immune modulators to alleviate inflammatory responses. Moreover, HSV-1 has the potential to carry endogenous enzymes involved in dopamine synthesis and therapeutic genes that protect neurons, thus restoring the functionality of cells damaged in Parkinson’s disease [236].

### Vaccine development and disease modeling

HSV, as a versatile tool carrier, is applied in various fields, including vaccine development and disease modeling. Through genetic engineering, HSV can express antigens specific to multiple viruses or bacteria, offering a novel method for concurrent vaccination against diverse pathogens. Its unique ability to infect both immune and nerve cells provides significant advantages for immune activation and neurological disease research. In the context of tumor vaccines, HSV vectors are used to deliver genes encoding tumor-specific antigens, such as DCs, eliciting robust immune responses that lead to effective tumor immunotherapy and long-lasting immune memory to prevent tumor relapse (Fig. 6b). Multiple vaccinations can further amplify the immune response and enhance the treatment and prevention outcomes. In addition, HSV carriers can be used to deliver Aβ vaccines, aiding in the prevention of brain Aβ plaque formation and clearance, thus providing a therapeutic approach for neurological conditions like Alzheimer’s disease [222, 237, 238].

As a gene vector, HSV can be used to construct disease models by expressing or knocking out specific genes. For example, HSV vectors can introduce mutated genes into neurons in animal models by carrying gene editing systems, such as CRISPR/Cas, to simulate the pathogenesis of neurodegenerative diseases such as Parkinson’s disease and Alzheimer’s disease (Fig. 6d). By carrying tumor-related or therapeutic genes, HSV can also induce tumorigenesis and assess the efficacy of tumor treatments in vivo. These models facilitate the study of tumor etiology, tumor cell biology, and tumor-immune system interactions, paving the way for innovative tumor diagnosis and treatment approaches. Additionally, HSV serves as a model for investigating viral infections and immune responses. By genetically modifying HSV vectors, researchers can introduce specific mutations or deletions to study virus-host interactions, viral replication and transmission mechanisms, and host immune reactions to viral infections. This modeling approach enhances our understanding of the pathological processes underlying viral infections and lays the foundation for the development of antiviral medications and vaccines [41, 239].

### Biological imaging and tracing

The ability to carry a larger genetic payload and efficiently amplify the host HSV-1 makes it a potentially powerful tool for imaging and tracing. Their neurotropic nature provides valuable insights into neuroscience (Fig. 6e) [10, 240–242]. The HSV-1 strain H129, with the unique feature of predominantly anterograde transneuronal transmission, represents a promising anterograde neuronal circuit tracer for mapping output neuronal pathways (Table 5). Over the years, the H129-derived anterograde tracing toolbox has expanded significantly, encompassing both polysynaptic and monosynaptic tracers labeled with various fluorescent proteins. These tracers have been instrumental in neuroanatomical studies that have revealed numerous critical neuronal circuits. One example is H129-G4, a polysynaptic tracer notable for its bright labeling intensity, which makes it ideal for mapping output networks. It stands out as the only anterograde transneuronal tracer compatible with fluorescence micro-optical sectioning tomography, enabling the decoding of whole-brain projections and neuronal morphology within a specific brain region. Nevertheless, it has limitations, such as a lack of starter cell specificity, potential retrograde labeling, and high toxicity. Another tracer, H129-dTK-tdT, is an anterograde monosynaptic tracer suitable for both specific and nonspecific tracing of starter neurons. However, it exhibits low labeling intensity and relatively high toxicity to starter neurons and requires immunostaining for visualization [4, 243].

**Table 5:** Anterograde neuronal tracers derived from HSV-1 (strain H129)

Candidate	Gene engineering	Function
H129-dTK-TT [1]	PCAG-tdTomato-TK cassette is inserted into the middle of the TK gene (*UL23*). The original TK is knocked out.	Red, polysynaptic tracer
H129-EGFP [3]	The EGFP is inserted between *UL26/26.5* and *UL27*.	Green, polysynaptic tracer
HSV-G4 [4, 5]	The BAC sequence is inserted between *UL22* and *UL23* to generate BAC-H129, and one EGFP is placed between the BAC sequence and UL23, and another is inserted between *US7* and *US8*.	Green, polysynaptic tracer
HSV-H8 [6]	The expression cassette containing GFP and AAV replicase is inserted between *lL37* and *UL38*.	Green, polysynaptic tracer
H129-dTK-tdT [5, 7]	The tdTomato expression cassette is inserted into BAC-H129 to replace the TK gene (*UL23*), resulting in TK deletion.	Red, monosynaptic tracer
H129-dTK-T2 [5]	Another identical tdTomato expression cassette is inserted into the genome of H129-dTK-tdT, between *US7* and *US8*.	Red, monosynaptic tracer
H129-dgK-G4 [10]	The gK gene (*UL53*) of H129-G4 is knocked out. It coinfects with an AAV helper that expresses gK.	Green, monosynaptic tracer

Although HSV-based biological imaging and tracing tools play a vital role in neuroscience, several concerns have arisen regarding these strategies. First, a prevalent issue with most current trans-neuronal tracers is whether they are strictly transmitted through synapses. The synaptic gap typically measures 20 nm; however, the average diameter of the H129 virion is approximately 200 nm, raising questions about how such a large viral particle can traverse such a narrow space [40, 76]. Second, the high toxicity and biological complexity of HSV often result in severe neuronal dysfunction or even death, posing a significant challenge [6]. Third, the large genome size of HSV-1 complicates DNA manipulation [244]. Further research characterizing HSV virology is imperative to optimize and develop novel HSV-based biological imaging and tracing tools.

Despite the vast potential of HSV as a tool carrier, it is crucial to approach its practical applications cautiously, owing to the inherent risks and challenges. First, HSV retains certain biosafety hazards even after modifications to reduce toxicity. Specifically, novel oHSVs that feature multigene mutations and are armed with specific foreign genes necessitate further research and investigation to enhance both their safety and efficiency. Furthermore, HSV tracers that possess strong toxicity have the potential to damage infected neurons, thereby preventing them from performing functional mapping. Second, the complexity of the HSV immune escape mechanism may alter the host immune response, possibly triggering inflammation and other complications. As for oHSV, it is necessary to find ways to diminish antiviral immunity while enhancing the virus's ability to trigger robust antitumor immunity. Third, the prolonged presence and replication of HSV vectors within the body could pose risks, such as gene mutations or cellular transformations. Additionally, the safety, stability, and targeting capabilities of the tool carriers have limitations. Although HSV exhibits neurotropism and dermatotropism, the HSV vector may infect target cells and other non-target cells, leading to nonspecific gene expression or adverse reactions. Therefore, improving the targeting ability of HSV vectors remains an important challenge in gene therapy. Finally, the key to tool vectors is the precise expression of target genes. However, currently, it is not possible to fully control the gene expression level and duration of HSV vectors in vivo, which may affect treatment efficacy and safety. In summary, although HSV has broad application prospects as an oncolytic virus and in gene therapy, it has many limitations. To overcome these limitations, it is necessary to continue in-depth research on the biological characteristics, pathogenesis, and interaction mechanisms between HSV and the host to better control its targeting and gene expression regulation ability in vivo and develop wider applications.

## Conclusions and perspectives

HSV, a lifelong disease affecting a large number of people of varying ages globally, is characterized by its complex pathogenesis. This review comprehensively discusses HSV's biological characteristics, infection cycle, and host-virus immune interplay. Notably, HSV serves as a “double-edged sword.” Its intricate pathogenesis and immune evasion have caused unprecedented disasters in humans, resulting in widespread and severe global infections, while hindering vaccine development.

Conversely, HSV remains a promising biological tool vital for disease and scientific research. This review highlights the recent advancements in HSV vaccine development and HSV-based tool applications, discussing their successes and challenges to guide future research.

Even though diverse strategies have been attempted, no ultimate cure or vaccine is yet available. The situation is mainly due to the intricate nature of HSV's pathogenesis and immune interplay, which acts as a significant obstacle to the comprehensive and profound understanding of the virus, thereby limiting the development of vaccines. To be specific, several vaccines face challenges, including a narrow therapeutic spectrum and limited effectiveness, which are constrained by the difficulty in identifying vaccine antigens capable of effectively inducing and maintaining robust immune responses. In addition to vaccine antigens, however, several limiting factors exist in vaccine development, such as viral culture systems, animal models, injection methods, and adjuvant use. Furthermore, as mentioned earlier, mRNA vaccines are a promising avenue because of their distinctive advantages, with initial studies of trivalent mRNA vaccines demonstrating superior efficacy compared to subunit formulations.

Despite these challenges, HSV has emerged as a promising biological tool due to its unique characteristics that play a pivotal role in disease and scientific research. As an OV, it effectively lyses tumor cells and recruits immune cells for anticancer therapy. Additionally, HSV serves as a versatile viral carrier, capable of transporting various foreign genes or therapeutic agents for gene and immune therapies. Equipped with marker genes, HSV functions as an imaging tool for enhanced visualization and tracking, particularly in neuroscience. However, practical applications involve numerous potential biological risks, including challenges in targeting specificity, gene expression stability, viral toxicity, lifelong latent infections, and achieving high precision during in vivo genome engineering. In summary, developing an effective vaccine and overcoming the limitations in its applications is a daunting task, and further research is urgently needed to comprehensively and deeply understand HSV.

Future advancements in HSV's life cycle, protein interactions, and immune evasion mechanisms will pave the way for effective HSV vaccines to prevent or mitigate infections. This includes identifying optimal antigens, overcoming technical hurdles in vaccine design and delivery, and assessing the efficacy and safety of candidate vaccines in clinical trials. Furthermore, this knowledge will also aid in optimizing HSV-based viral vectors for gene therapy by enhancing targeting specificity, maintaining stable gene expression, minimizing viral toxicity, and achieving high precision during in vivo genome engineering. HSV's oncolytic potential can be boosted by enhancing its tumor selectivity, immune response induction, and developing combination therapies with other immunomodulatory agents. Moreover, HSV's potential as a neuroimaging tool can be augmented by developing more sensitive and specific marker genes, improving imaging resolution, and expanding the scope of neurological disorders that can be studied. In summary, HSV research holds great promise for both addressing public health challenges and advancing scientific knowledge. By addressing the current limitations and exploring new research avenues, we can gain control over HSV infection and harness its full potential as a tool for improving human health.

## Data Availability

Not applicable.
